# Dual Actions of Butyrate on Immunoepithelial Remodeling in Ex Vivo Intestinal Biopsies from Patients with Inflammatory Bowel Disease

**DOI:** 10.3390/metabo16060395

**Published:** 2026-06-06

**Authors:** Sara Troisi, Cesare Pane, Letizia Masi, Filippo Biamonte, Domenico Scannone, Antonio Cappa Spina, Miriam Di Mattia, Valeria Emoli, Ivan Capobianco, Sonia Distante, Barbara Rondinone, Valentina Petito, Antonio Gasbarrini, Loris Riccardo Lopetuso, Franco Scaldaferri

**Affiliations:** 1 CeMAD Translational Research Laboratories, Digestive Disease Center (CeMAD), Department of Medical and Surgical Sciences, Fondazione Policlinico Universitario “A. Gemelli” IRCCS, 00168 Rome, Italy; sara.troisi02@icatt.it (S.T.); cesare.pane@guest.policlinicogemelli.it (C.P.); letizia.masi@policlinicogemelli.it (L.M.); filippobimo@libero.it (F.B.); valeria.emoli@policlinicogemelli.it (V.E.); sonia.distante01@icatt.it (S.D.); barbara.rondinone@unicatt.it (B.R.); antonio.gasbarrini@unicatt.it (A.G.); franco.scaldaferri@policlinicogemelli.it (F.S.); 2 Department of Translational Medicine and Surgery, Università Cattolica del Sacro Cuore, 00168 Rome, Italy; antonio.cappaspina01@icatt.it (A.C.S.); ivan.capobianco01@icatt.it (I.C.); 3 Histopathology Unit, Dipartimento di Scienze della Salute della Donna, del Bambino e di Sanità Pubblica, Fondazione Policlinico Universitario “A. Gemelli” IRCCS, 00168 Rome, Italy; domenico.scannone@unicatt.it; 4 Center for Advanced Studies and Technology (CAST), Department of Medicine and Aging Science, G. D’Annunzio University of Chieti-Pescara, 66100 Chieti, Italy; miriam.dimattia@unich.it; 5 IBD Unit, Digestive Disease Center (CeMAD), Department of Medical and Surgical Sciences, Fondazione Policlinico Universitario “A. Gemelli” IRCCS, 00168 Rome, Italy; lorisriccardo.lopetuso@guest.policlinicogemelli.it; 6 Department of Life Science, Health, and Health Professions, Link Campus University, 00168 Rome, Italy

**Keywords:** butyrate, inflammatory bowel disease, dose-dependent effects, ex vivo, cytokines, epithelial morphology

## Abstract

**Background/Objectives:** Butyrate, a microbiota-derived short-chain fatty acid, plays a central role in intestinal homeostasis, yet its concentration-dependent effects on human inflamed mucosa remain poorly defined. This study investigates the dose-dependent impact of butyrate on inflammatory signaling and epithelial architecture in ex vivo intestinal biopsies from patients with inflammatory bowel disease (IBD), including ulcerative colitis (UC) and Crohn’s disease (CD). **Methods:** Intestinal biopsies from six IBD patients (four UC, two CD) were cultured ex vivo for 24 h with calcium butyrate (5, 10, 100 mM). Cytokine secretion was analyzed by multiplex immunoassay, total protein release was quantified, and glandular morphology was assessed by stereological analysis. **Results:** Butyrate was associated with a dose-dependent reduction in pro-inflammatory cytokines-including TNF-α, IFN-γ, IL-6, and IL-17-in both inflamed and non-inflamed tissue. Non-linear responses were observed for specific mediators, such as IP-10, which displayed a biphasic pattern in inflamed UC biopsies. Notably, UC and CD biopsies exhibited distinct response profiles: UC samples showed marked cytokine modulation, including reduction in IL-15, whereas CD samples, evaluated at 5 and 10 mM, showed limited modulation across conditions. A trend toward reduced total protein secretion was observed in diseased UC biopsies following butyrate exposure. Stereological analysis revealed preservation of glandular area at 5 mM, reduction at 10 mM, and extensive tissue disruption at 100 mM precluding structural evaluation. **Conclusions:** In this pilot study, butyrate exerts dose-dependent and disease-specific effects in human IBD mucosa within a narrow therapeutic window. These exploratory findings suggest that dose-optimized strategies for butyrate-based interventions may be particularly relevant in UC, although larger confirmatory studies are needed.

## 1. Introduction

Inflammatory bowel disease (IBD), encompassing Crohn’s disease (CD) and ulcerative colitis (UC), comprises a group of chronic relapsing inflammatory disorders characterized by dysregulated immune responses, impaired epithelial barrier integrity, and altered host–microbiota interactions [1]. Epithelial barrier dysfunction is increasingly recognized not merely as a secondary consequence of inflammation, but as an early pathogenic event contributing to disease initiation and perpetuation [2]. When barrier function is compromised, increased intestinal permeability facilitates aberrant immune activation and establishes a self-reinforcing cycle of inflammation, epithelial injury, and defective mucosal repair [3].

Among microbial-derived metabolites, short-chain fatty acids (SCFAs)—primarily acetate, propionate, and butyrate—are produced through bacterial fermentation of dietary fibers in the colon and exert profound effects on epithelial metabolism and immune regulation [4]. Beyond serving as metabolic substrates, SCFAs participate in substrate-dependent immune remodeling by influencing T cell differentiation, macrophage polarization, dendritic cell activity, and inflammatory cytokine production. These effects collectively contribute to intestinal immune homeostasis and epithelial barrier reinforcement [5,6,7]. Butyrate serves as the principal metabolic substrate for colonocytes and exerts immunomodulatory effects through inhibition of histone deacetylases and activation of G-protein-coupled receptors, suppressing pro-inflammatory cytokine production and reinforcing epithelial barrier function [8,9].

Perturbations of the intestinal microbiota represent a hallmark of IBD and are frequently associated with a reduction in butyrate-producing bacteria, including *Faecalibacterium prausnitzii* and *Roseburia hominis*, accompanied by decreased luminal SCFA concentrations [10]. Based on these observations, butyrate has been proposed as a therapeutic strategy for IBD. A recent prospective observational study demonstrated that calcium butyrate combined with probiotics and fructooligosaccharides achieved therapeutic success in 95% of patients with UC in remission when added to 5-ASA therapy, compared to 57% with 5-ASA alone [11]. However, the biological effects of butyrate appear strongly concentration-dependent: while physiological concentrations support epithelial metabolism and anti-inflammatory signaling, higher concentrations may induce metabolic stress or cytotoxicity [12].

A limitation of many studies investigating butyrate biology is the reliance on simplified in vitro systems or animal models [13]. Ex vivo culture models based on freshly isolated intestinal biopsies preserve epithelial architecture and maintain interactions between epithelial, stromal, and immune cell compartments, enabling assessment of pharmacological interventions directly in human tissue [14,15]. Furthermore, quantitative stereological approaches offer unbiased evaluation of structural changes in epithelial architecture that may not be captured by conventional histological assessment [16].

Despite the recognized biological relevance of butyrate, the dose-dependent impact on cytokine secretion and structural epithelial responses within inflamed human mucosa has not been systematically investigated. In the present pilot study, we employed an ex vivo biopsy model to examine the concentration-dependent effects of butyrate on inflamed and non-inflamed human intestinal mucosa, integrating inflammatory, secretory, and structural readouts to provide a comprehensive evaluation of butyrate activity at the tissue level. We further explored whether these responses differ between ulcerative colitis and Crohn’s disease, with the aim of generating preliminary hypotheses regarding disease-specific differences in tissue-level responses.

## 2. Materials and Methods

### 2.1. Study Population and Biopsy Collection

Intestinal biopsies were obtained from patients with inflammatory bowel disease undergoing ileocolonoscopy for persistent or recurrent mucosal inflammation at the Centro Malattie Apparato Digerente (CeMAD), Fondazione Policlinico Universitario “A. Gemelli” IRCCS, Rome, Italy. The study included six patients (*n* = 6): four with ulcerative colitis (UC) and two with Crohn’s disease (CD). Enrolled subjects had a mean age of 30 ± 5 years (range: 24–36), disease duration < 7 years, and a history of failure to first-or second-line biologic therapy.

The UC subgroup showed moderate-to-severe endoscopic activity, as assessed by the Mayo endoscopic score (mean score 2.7 ± 0.6; median 3; range 2–3). Histological reports confirmed active colitis/proctocolitis, with chronic-active inflammation, cryptitis, erosive changes, and architectural distortion. UC patients had exposure to conventional therapy and advanced biologic agents, including anti-TNF, anti-integrin, anti-IL-12/23, or anti-IL-23 therapies, with optimization or therapeutic switch in selected cases.

CD patients included one biologic-experienced subject with active ileocolonic disease despite anti-IL-12/23 therapy, documented by Simple Endoscopic Score for Crohn’s Disease (SES-CD; score 15) and one postoperative subject with endoscopic recurrence after ileocecal resection, documented by Rutgeerts i2, evaluated before initiation of postoperative biologic therapy. CD histology documented active ileal inflammation or postoperative inflammatory recurrence. Overall, the cohort reflected patients with active, difficult-to-control IBD rather than quiescent disease.

For each patient, paired biopsies were collected from inflamed mucosa and macroscopically healthy mucosa, when available. Immediately after collection, biopsies were transferred into sterile culture medium consisting of Dulbecco’s modified Eagle’s medium (DMEM; Sigma-Aldrich, St. Louis, MO, USA) supplemented with 10% fetal bovine serum (FBS; Sigma-Aldrich, St.Louis, MO, USA) and 1% penicillin–streptomycin (Sigma-Aldrich, St.Louis, MO, USA) and processed for ex vivo organ culture experiments.

All patients provided written informed consent. The study was approved by the Ethics Committee of the Università Cattolica del Sacro Cuore, Rome, Italy (protocol number ID 3708; approval date: 25 March 2024).

### 2.2. Ex Vivo Culture of Intestinal Biopsies

Biopsies were washed with phosphate-buffered saline (PBS; Sigma-Aldrich, St.Louis, MO, USA), gently blotted to remove excess liquid, weighed, and immediately placed in culture. Tissue samples were cultured for 24 h on 30 μm PET track-etched membranes in 24-well plates (VWR International PBI S.r.l., Milan, Italy). Biopsies were maintained in culture medium under standard culture conditions (37 °C, 5% CO_2_) and exposed to calcium butyrate (Dicofarm, Rome, Italy) at different concentrations.

Specimens were incubated either in control medium or in culture medium supplemented with calcium butyrate at 5 mM, 10 mM, or 100 mM. The 5 mM and 10 mM concentrations were selected to approximate physiological luminal butyrate levels in the human colon [17]. The 100 mM concentration was included as a supraphysiological dose to explore potential cytotoxic effects and define the upper limit of the therapeutic window in ex vivo tissues.

After 24 h of incubation, culture supernatants were collected and stored at −80 °C for subsequent analysis of cytokine and protein secretion. Following supernatant collection, the biopsies were embedded in optimal cutting temperature (OCT) compound (Diapath S.p.A., Martinengo, Italy) and stored at −80 °C for downstream stereological analyses.

### 2.3. Quantification of Total Protein Secretion

Total protein concentrations in culture supernatants were determined using the Pierce™ Bradford Protein Assay Kit (Thermo Fisher Scientific, Waltham, MA, USA) according to the manufacturer’s instructions. Briefly, aliquots of collected supernatants were incubated with Bradford reagent, and absorbance was measured spectrophotometrically. Protein concentrations were calculated by interpolation from a standard calibration curve generated using bovine serum albumin (BSA) standards (Bio-Rad Laboratories, Hercules, CA, USA) and expressed as µg/mL. This analysis was performed to assess the overall secretory activity of intestinal biopsies following exposure to calcium butyrate.

### 2.4. Cytokine Profiling

Cytokine secretion from cultured intestinal biopsies was quantified using a multiplex bead-based immunoassay. Culture supernatants collected after 24 h of incubation were analyzed using the Bio-Plex Pro™ Human Cytokine 27-plex Assay (Bio-Rad Laboratories, Hercules, CA, USA) according to the manufacturer’s instructions. Data acquisition was performed using the Bio-Plex^®^ 200 system (Bio-Rad Laboratories, Hercules, CA, USA) and cytokine concentrations were calculated from five-parameter logistic (5-PL) standard curves generated with Bio-Plex Manager software (version 6.2; Bio-Rad Laboratories, Hercules, CA, USA). Results were expressed as pg/mL and normalized to biopsy wet weight.

### 2.5. Stereological Analysis of Epithelial Gland Architecture

Morphological changes in intestinal epithelial architecture following butyrate exposure were quantified using a design-based stereological approach. Biopsies previously embedded in OCT compound were cryosectioned at a thickness of 3 µm and analyzed using the Stereo Investigator software platform (version 2024.1.3; MBF Bioscience, Williston, VT, USA). Selected sections were stained with haematoxylin and eosin (H&E) for morphological visualization of glandular structures (Leica Microsystems, Wetzlar, Germany).

For each biopsy, five non-consecutive sections were analyzed, and 25 glandular profiles per section were quantified. Sections were selected using a systematic uniform random sampling strategy to reduce selection bias. Regions of interest (ROIs) were manually delineated to include the epithelial mucosal compartment containing identifiable glandular/crypt structures, while excluding areas with sectioning artifacts, tissue folding, detachment, or severe tissue disruption. The same ROI selection criteria were applied across all experimental conditions. The 100 mM butyrate condition was excluded from quantitative stereological analysis when severe tissue damage and loss of glandular architecture precluded reliable gland identification. Analyses were performed in a blinded manner with respect to treatment condition. Interobserver reproducibility was assessed in a subset of samples, showing high concordance, with an intraclass correlation coefficient > 0.85. The Cavalieri estimator probe included in the Stereo Investigator software was applied to obtain an unbiased quantitative estimate of the glandular (crypt) area.

A systematic point grid was subsequently superimposed on the outlined structures, and the estimated glandular area (µm^2^) was calculated based on the number of grid points intersecting the structure according to the Cavalieri principle.

### 2.6. Statistical Analysis

Statistical analyses were performed using GraphPad Prism software (version 10, San Diego, CA, USA). Data distribution was assessed using the Shapiro–Wilk test to evaluate normality. Continuous variables are presented as mean ± SD unless otherwise specified.

Inflamed and non-inflamed biopsies collected from the same patients were analyzed as paired samples throughout, using paired tests where applicable. For treatment comparisons within each tissue condition, repeated-measures one-way ANOVA was used for normally distributed data, followed by corrected post hoc multiple-comparison tests, as appropriate. When normality assumptions were not met, the Friedman test followed by Dunn’s multiple-comparison test was applied. Direct comparisons between matched inflamed and non-inflamed biopsies were performed using paired Student’s *t*-test for normally distributed data or Wilcoxon matched-pairs signed-rank test for non-normally distributed data. Multiple comparisons were corrected using Tukey’s or Dunnett’s post hoc test for parametric analyses, or Dunn’s test for non-parametric analyses, according to the comparison performed. Given the very small sample size of the CD subgroup (*n* = 2) and of the stereological analysis (*n* = 2), no inferential statistical testing was performed for these datasets, which are presented descriptively. A *p* value < 0.05 was considered statistically significant.

## 3. Results

### 3.1. Butyrate Dynamically Modulates Cytokine Secretion in Ex Vivo Cultured IBD Biopsies

To investigate the immunomodulatory effects of butyrate on the intestinal mucosa, cytokine secretion was quantified in supernatants collected from ex vivo cultured colonic biopsies obtained from patients with ulcerative colitis. Analyses were performed on paired biopsies obtained from inflamed and macroscopically non-inflamed areas of the same patients. Both macroscopically non-inflamed (healthy tissue) and inflamed (diseased tissue) areas were cultured for 24 h in the presence of control medium or increasing concentrations of butyrate (5 mM, 10 mM, 100 mM), followed by multiplex cytokine profiling.

Overall, butyrate exposure resulted in dose-dependent modulation of inflammatory mediators, with distinct patterns observed between healthy and inflamed tissues.

**Figure 1:**
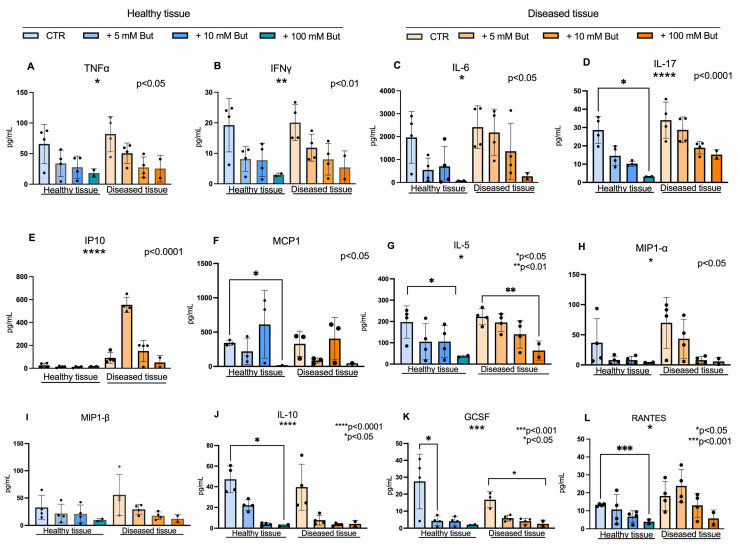
Butyrate reshapes the inflammatory cytokine landscape in ex vivo cultured IBD biopsies. Cytokine secretion was measured in supernatants from ex vivo cultured intestinal biopsies obtained from healthy (macroscopically non-inflamed) and diseased (inflamed) mucosal areas of UC patients (*n* = 4) after 24 h incubation with control medium (CTR) or butyrate (5, 10, and 100 mM). Multiplex analysis revealed a dose-dependent modulation of inflammatory mediators, including TNF-α (**A**), IFN-γ (**B**), IL-6 (**C**), IL-17 (**D**), (IP-10 (**E**), MCP-1 (**F**), IL-5 (**G**), MIP-1α (**H**), MIP-1β (**I**), IL-10 (**J**), G-CSF (**K**), and RANTES (**L**). Diseased biopsies showed higher baseline cytokine secretion, which was broadly attenuated following butyrate exposure. Data are expressed as pg/mL (mean ± SD). Individual dots represent biological replicates. Treatment effects within each tissue condition were analyzed using repeated-measures one-way ANOVA for normally distributed data or Friedman test for non-normally distributed data, followed by corrected post hoc comparisons. Matched inflamed and non-inflamed biopsies from the same patients were analyzed using paired tests. Normality was assessed by Shapiro–Wilk test. Statistical significance is indicated as * *p* < 0.05, ** *p* < 0.01, *** *p* < 0.001, **** *p* < 0.0001.

In non-inflamed mucosal biopsies, butyrate treatment induced consistent, dose-dependent reduction in pro-inflammatory cytokines. Specifically, secretion of TNF-α, IFN-γ, IL-6, and IL-17 progressively decreased with increasing butyrate concentrations compared with control conditions (*p* < 0.05) (Figure 1A–D). A similar suppressive trend was observed for chemokines involved in leukocyte recruitment, including MIP1-alpha and RANTES, which were significantly reduced following butyrate exposure (Figure 1H,L). Notably, the highest concentration tested (100 mM) produced the most pronounced inhibitory effects across multiple cytokines.

Inflamed UC biopsies displayed higher baseline cytokine secretion compared with non-inflamed tissue, reflecting the ongoing inflammatory state. Despite the elevated baseline levels, butyrate treatment markedly attenuated the production of key inflammatory mediators in a pattern comparable to that observed in non-inflamed tissue. Significant reductions were observed for TNF-α, IFN-γ, IL-17, and IL-6, confirming broad anti-inflammatory effects across tissue states (Figure 1A–D). In parallel, butyrate also modulated chemokine secretion, including MIP-1α, MCP-1, and RANTES, suggesting a potential impact on immune cell recruitment pathways (Figure 1F,H,L).

Two cytokines exhibited distinct, non-linear response patterns. The chemokine IP-10 (CXCL10), which showed minimal levels in non-inflamed tissue but markedly elevated baseline secretion in inflamed biopsies, displayed a biphasic response to butyrate exposure. The 5 mM concentration induced a pronounced increase compared with control conditions (*p* < 0.0001), followed by progressive decline at higher concentrations (10 mM and 100 mM), indicating complex, concentration-dependent regulation (Figure 1E).

Additionally, IL-10 secretion, particularly elevated in inflamed tissue, was significantly reduced with increasing butyrate concentrations (*p* < 0.0001). This unexpected reduction in an anti-inflammatory cytokine suggests broader remodeling of the cytokine network rather than simple anti-inflammatory polarization (Figure 1J).

Consistent with these findings, G-CSF and IL-5 were also significantly modulated by butyrate exposure, further supporting the ability of this microbial metabolite to influence multiple immune pathways within the intestinal mucosa (Figure 1G,K).

### 3.2. Preliminary Evidence of Differential Cytokine Responses Between UC and CD Biopsies

**Figure 2:**
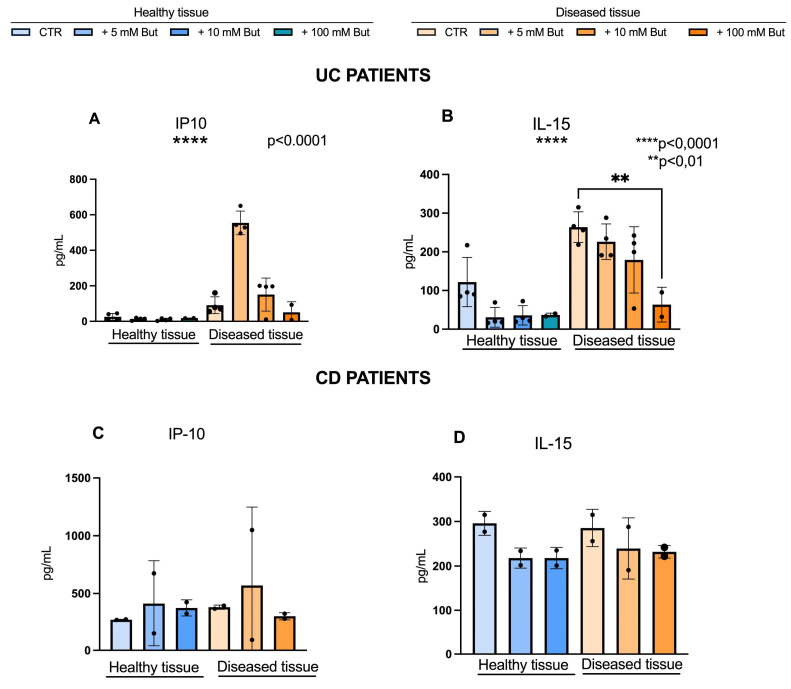
Effect of butyrate on cytokine secretion from healthy and diseased biopsies of UC and CD patients. (**A**,**B**): Cytokine secretion (IP-10 and IL-15) in supernatants from healthy (macroscopically non-inflamed) and diseased (inflamed) biopsies of UC (*n* = 4) patients after 24 h of incubation with control medium (CTR) or butyrate at 5, 10, and 100 mM concentrations. (**C**,**D**): Cytokine secretion (IP-10 and IL-15) in supernatants from healthy and diseased biopsies of CD (*n* = 2) patients after 24 h of incubation with control medium (CTR) or butyrate at 5 and 10 mM concentrations. Data are presented as mean ± SD, and individual dots represent biological replicates. For UC biopsies, treatment effects within each tissue condition were analyzed using repeated-measures one-way ANOVA for normally distributed data or Friedman test for non-normally distributed data, followed by corrected post hoc comparisons. Matched inflamed and non-inflamed biopsies from the same patients were analyzed using paired tests when directly compared. Normality was assessed by Shapiro–Wilk test. For CD biopsies, no inferential statistical analysis was performed because of the limited sample size, and data are presented descriptively. Significance is indicated as ** *p* < 0.01 and **** *p* < 0.0001.

To assess whether the cytokine response to butyrate differed between IBD subtypes, analyses were performed separately in biopsies derived from patients with ulcerative colitis and Crohn’s disease. In UC biopsies, diseased mucosal samples showed markedly higher basal secretion of several cytokines compared with healthy tissue. IP-10 levels were strongly increased in diseased UC biopsies compared to healthy tissue (*p* < 0.0001). Butyrate treatment at 5 mM resulted in a further increase in IP-10 secretion, whereas higher concentrations progressively suppressed its levels, with the most pronounced decrease observed at 100 mM butyrate (Figure 2A). A similar pattern was observed for IL-15, which was significantly elevated in diseased UC tissue compared with healthy mucosa. Butyrate exposure resulted in a progressive reduction in IL-15 levels across increasing concentrations, with significant suppression observed at 100 mM butyrate (*p* < 0.01) (Figure 2B).

In contrast, CD biopsies displayed a substantially different cytokine profile. Baseline levels of both IP-10 and IL-15 were relatively comparable between healthy and diseased CD samples, and butyrate treatment did not induce clear dose-dependent modulation. Cytokine levels remained largely stable across treatment conditions (Figure 2C,D). These preliminary results suggest distinct cytokine response patterns between UC and CD biopsies following butyrate exposure, with UC samples exhibiting a more pronounced modulation of inflammatory mediators. However, given the limited CD subgroup size (*n* = 2), these observations should be interpreted with caution and considered hypothesis-generating rather than conclusive.

### 3.3. Butyrate Induces a Trend Toward Reduced Secretory Activity in Diseased Intestinal Biopsies

To evaluate the metabolic impact of butyrate on intestinal tissue, total protein secretion was quantified in culture supernatants from UC and CD biopsies following 24 h exposure to increasing butyrate concentrations.

**Figure 3:**
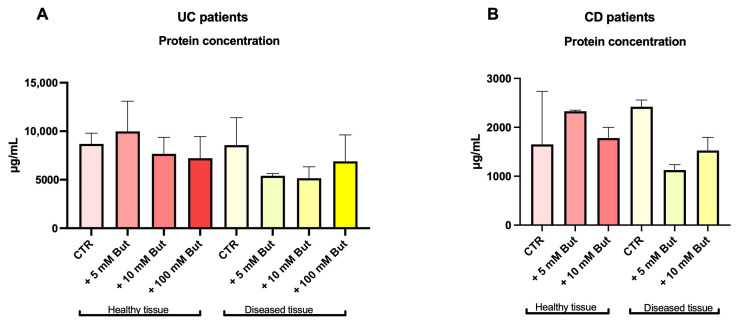
Effect of butyrate on total protein secretion from healthy and diseased biopsies of UC and CD patients. (**A**): Total protein concentration in supernatants from healthy (macroscopically non-inflamed) and diseased (inflamed) biopsies of UC (*n* = 4) patients after treatment with control medium (CTR) or butyrate at 5 mM, 10 mM, and 100 mM concentrations for 24 h. (**B**): Total protein concentration in supernatants from healthy and diseased biopsies of CD (*n* = 2) patients after treatment with control medium (CTR) or butyrate at 5 mM and 10 mM concentrations for 24 h. Data are expressed as mean ± SD. In UC biopsies, repeated-measures one-way ANOVA or Friedman test was applied according to Shapiro–Wilk normality testing, followed by corrected post hoc comparisons; no statistically significant differences were observed. For CD biopsies, no inferential statistical analysis was performed because of the limited sample size, and data are presented descriptively.

In UC biopsies, diseased tissue showed slightly higher baseline protein secretion compared to healthy tissue. Although no statistically significant differences were observed, butyrate treatment appeared to induce a modest reduction in protein secretion in diseased tissue at 5 mM and 10 mM concentrations, suggesting a trend toward decreased secretory activity. At 100 mM, protein secretion levels in diseased tissue were comparable to control conditions, possibly indicating a plateau effect at higher concentrations. (Figure 3A).

In contrast, CD biopsies exhibited lower baseline protein secretion in diseased tissue compared to UC. Although no statistically significant differences were observed, butyrate treatment appeared to induce a modest reduction in protein secretion in diseased tissue at 5 mM, though this effect was not maintained at 10 mM. Of note, 100 mM butyrate was not assessed in this cohort (Figure 3B).

Overall, butyrate treatment appeared to induce a trend toward reduced protein secretion in diseased biopsies, more appreciable in UC than in CD biopsies, although no statistically significant differences were observed in either cohort.

### 3.4. Stereological Analysis Reveals Dose-Dependent Effects of Butyrate on Glandular Architecture

To evaluate the structural impact of butyrate on intestinal tissue, stereological analysis was performed to measure gland area in healthy and diseased biopsies following 24 h exposure to butyrate.

In healthy tissue, treatment with 5 mM butyrate did not induce appreciable changes in gland area compared to control conditions. Treatment with 10 mM butyrate appeared to reduce gland area, though this effect did not reach statistical significance.

**Figure 4:**
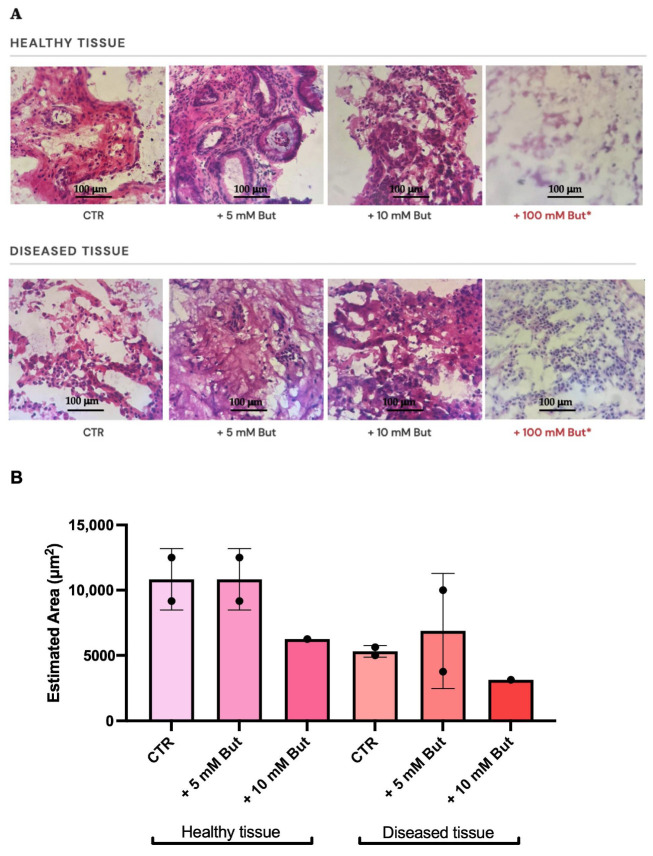
Stereological evaluation of gland area in colonic biopsies exposed to increasing concentrations of butyrate. (**A**). Representative H&E-stained sections from one UC patient showing colonic glands in healthy and diseased biopsies treated with control medium (CTR) or calcium butyrate at 5, 10, and 100 mM for 24 h. Images were uniformly adjusted for white balance and brightness to correct for inter-sample staining variability. Scale bar = 100 µm. * 100 mM butyrate treatment resulted in severe tissue damage with loss of glandular architecture, precluding stereological analysis. (**B**). Estimated gland area (µm^2^) was measured using a stereological approach on healthy and diseased biopsies of UC patients treated with CTR or butyrate at 5 mM and 10 mM concentrations for 24 h. Data represent mean ± SD of gland areas averaged per biopsy. Given the small sample size (*n* = 2), no inferential statistical testing was performed, and results are presented descriptively.

In diseased tissue, overall gland areas were smaller compared to healthy tissue, as expected due to the presence of inflammation and epithelial damage. Butyrate exposure at 5 mM resulted in a slight trend toward increased gland area, suggesting a potential reparative effect at lower concentrations. However, 10 mM butyrate caused a similar reduction in gland area as observed in healthy tissue, suggesting that inflamed mucosa is more susceptible to higher concentrations of butyrate (Figure 4A,B). Notably, stereological evaluation could not be performed on biopsies exposed to 100 mM butyrate due to extensive tissue disruption and loss of recognizable glandular architecture (Figure 4A).

These observations reflect morphological assessment only; no formal cytotoxicity assays were conducted.

These data suggest that butyrate modulates glandular architecture in a dose-dependent manner, with 5 mM butyrate slightly enhancing glandular area in diseased tissue and 10 mM butyrate reducing gland area in both healthy and diseased tissues. However, no statistically significant effects were observed.

## 4. Discussion

Butyrate, a four-carbon short-chain fatty acid produced by microbial fermentation of dietary fiber, is a critical metabolite for intestinal homeostasis. In inflammatory bowel disease, both butyrate-producing bacteria and fecal butyrate levels are significantly reduced, and impaired butyrate metabolism has been documented in UC mucosa [18].Despite extensive evidence of butyrate’s anti-inflammatory effects in cellular models [19,20], its concentration-dependent impact on intact human intestinal mucosa remains poorly defined, and direct comparisons between ulcerative colitis and Crohn’s disease at the tissue level are lacking. Here, we address this gap using an ex vivo human biopsy model that preserves mucosal architecture and enables integrated assessment of immunological, secretory, and structural responses, with glandular architecture quantified through a novel stereological approach. Our findings demonstrate that butyrate exerts dose-dependent effects within a narrow concentration range: intermediate concentrations (5–10 mM) promote anti-inflammatory responses, whereas supraphysiological exposure (100 mM) induces cytotoxicity and tissue damage. Notably, these responses appear to differ between ulcerative colitis and Crohn’s disease, although this observation remains preliminary given the limited sample size.

Butyrate significantly reduced the secretion of key pro-inflammatory cytokines, including TNF-α, IFN-γ, IL-6, and IL-17, in both non-inflamed and inflamed UC biopsies in a dose-dependent manner. These findings are consistent with recent mechanistic studies suggesting that histone deacetylase 3 (HDAC3) inhibition in intestinal monocytes and macrophages may represent an important anti-inflammatory mechanism of butyrate in IBD tissues [21,22,23], although direct assessment of HDAC activity was not performed in the present study. Importantly, our data extend these observations beyond isolated immune cell systems, demonstrating that butyrate can coordinately modulate inflammatory signaling within the complex multicellular architecture of human intestinal tissue. Beyond cytokine suppression, butyrate also reduced the secretion of key chemokines, including MCP-1, MIP-1α, and RANTES, suggesting a broader impact on leukocyte recruitment. This coordinated downregulation of both cytokine- and chemokine-mediated pathways is consistent with the hypothesis that butyrate may broadly modulate the inflammatory network at the tissue level [9,24], although the precise cellular and molecular mechanisms underlying these observations remain to be defined.Notably, two cytokines exhibited non-linear behavior. IP-10 (CXCL10) displayed a biphasic pattern in inflamed UC biopsies, with transient elevation at 5 mM followed by suppression at higher doses, potentially suggesting differential sensitivity of distinct mucosal cell populations. IL-10 levels were also unexpectedly reduced, suggesting broader cytokine network remodeling rather than simple anti-inflammatory polarization.

This finding should be interpreted cautiously, as IL-10 reduction may reflect an overall attenuation of inflammatory signaling and compensatory anti-inflammatory responses rather than selective suppression of protective pathways. Alternatively, concentration-dependent effects on IL-10-producing mucosal cells cannot be excluded. This interpretation is consistent with recent transcriptomic, metabolomic, and microbiome-based studies showing that microbiota-derived signals and probiotic interventions can coordinately modulate inflammatory cytokine networks, epithelial barrier integrity, microbial composition, and host metabolic pathways during intestinal inflammation and infection [25,26]. In line with this concept, microbial-derived butyrate has been shown to promote colonic regulatory T-cell differentiation and to contribute to intestinal immune homeostasis, supporting the view that SCFA-mediated effects involve integrated immune-metabolic remodeling rather than isolated cytokine suppression [27]. Since cell-specific viability and functional assays were not performed, this observation remains exploratory and requires further mechanistic validation.

An exploratory observation of this pilot study is the apparent differential cytokine response to butyrate between UC and CD biopsies.

UC samples exhibited pronounced modulation of IP-10 and IL-15 following butyrate exposure, whereas CD biopsies showed relatively stable cytokine levels across treatment conditions.

Although this pattern may reflect distinct immunopathological features of the two diseases, including superficial mucosal inflammation and epithelial barrier dysfunction in UC and transmural inflammation with Th1/Th17 predominance in CD [28,29], these findings should be interpreted with caution due to the very small CD subgroup and potential inter-patient variability.

While meta-analyses have documented distinct SCFA alterations between UC and CD, with UC patients showing more pronounced reductions in propionate and acetate [30], our pilot study provides preliminary tissue-level evidence that exogenous butyrate may differentially modulate cytokine responses in human IBD biopsies.

The marked reduction in IL-15 in UC biopsies is particularly relevant: IL-15 is highly expressed in inflamed IBD mucosa, promotes inflammatory Th17 cells in the intestine, and its overexpression has been associated with intestinal dysbiosis and butyrate deficiency [31,32,33]. The pronounced IL-15 suppression observed in UC but not CD biopsies represents a novel exploratory finding that may support the future development of disease-specific metabolite-based interventions.

In addition, butyrate induced a consistent trend toward reduced total protein secretion, particularly at 5–10 mM, more evident in UC than in CD, further reinforcing the concept of differential metabolic responsiveness—though this finding did not reach statistical significance in either cohort.

A key methodological innovation of this study is the application of stereological analysis to quantify glandular architecture in human IBD tissue following metabolite exposure. The “butyrate paradox”—whereby physiological concentrations support colonocyte metabolism while supraphysiological concentrations inhibit proliferation and induce cellular stress [12,34,35]—has been extensively characterized at the molecular level, but no prior work has provided a structural assessment in human tissue.

From a metabolic perspective, intermediate butyrate concentrations may support epithelial homeostasis by fueling colonocyte mitochondrial oxidative metabolism and ATP production, thereby contributing to redox balance, barrier integrity, and mucosal repair. Conversely, supraphysiological exposure may overwhelm epithelial metabolic capacity, particularly in inflamed mucosa with impaired butyrate oxidation, promoting mitochondrial stress, oxidative imbalance, and cytotoxic injury [36,37,38].

Our stereological data revealed a trend toward glandular preservation at 5 mM and reduction at 10 mM, without reaching statistical significance. Most notably, tissue exposed to 100 mM butyrate could not be evaluated due to extensive structural disruption—an unambiguous qualitative finding that underscores the narrow margin between therapeutic and cytotoxic concentrations, which may be further narrowed in UC by impaired mucosal butyrate oxidation [39]. The specific mechanisms underlying this structural damage, including apoptosis, oxidative stress, or metabolic failure, were not directly assessed and warrant future investigation.

Together, these findings support the translational relevance of targeting microbial metabolite availability to modulate mucosal inflammation and epithelial integrity [40,41], while our ex vivo human biopsy model provides complementary tissue-level evidence that butyrate responsiveness is concentration-dependent and may differ between UC and CD.These findings have important implications for butyrate-based interventions in IBD. The robust cytokine suppression at 5–10 mM is consistent with recent clinical evidence showing that butyrate supplementation reduced inflammatory markers and improved quality of life in active UC patients [11,42,43]. In contrast, the limited responsiveness in CD and the structural damage at 100 mM suggest that both disease subtype and dose selection are critical determinants of therapeutic outcome, and warrant careful consideration in the design of future clinical strategies.

This study has several strengths, including the use of freshly isolated human biopsies that preserve mucosal architecture and cellular interactions, comprehensive assessment of cytokine secretion, total protein output, and the novel application of stereological methods to this context. Importantly, the use of paired biopsies from inflamed and non-inflamed mucosa within the same patients minimizes inter-individual variability and enables a more controlled assessment of butyrate-induced effects at the tissue level.

Nevertheless, several important limitations must be acknowledged. The most critical is the small sample size (*n* = 6 total; UC = 4, CD = 2), which substantially limits statistical power across all endpoints and precludes definitive conclusions. The CD subgroup is particularly underpowered, and the absence of statistically significant dose-dependent cytokine modulation in CD biopsies may reflect true biological differences but could equally result from insufficient sample size and high inter-patient variability. Accordingly, all findings—and especially the UC vs. CD comparisons—should be regarded as exploratory and hypothesis-generating. Additionally, the absence of dedicated viability assays precludes definitive interpretation of the protein secretion data, as alternative explanations such as metabolic suppression or reduced tissue viability cannot be excluded. Finally, the short-term nature of ex vivo culture does not capture dynamic host–microbiota interactions or long-term metabolite effects occurring in vivo. Future adequately powered studies are required to validate these findings and define the temporal dynamics of butyrate-induced effects on mucosal homeostasis.

## 5. Conclusions

This pilot study provides preliminary evidence that butyrate exerts broad, concentration-dependent effects on inflammatory signaling and epithelial architecture in human intestinal tissue, with a narrow therapeutic window between beneficial and cytotoxic concentrations. The observed differences in cytokine responses between UC and CD biopsies suggest potentially disease-specific pathophysiology that may influence the efficacy of metabolite-based therapeutic approaches, though these observations must be interpreted cautiously given the limited sample size and the exploratory nature of the study. These hypothesis-generating findings support further investigation into butyrate signaling pathways as a strategy to restore intestinal homeostasis in IBD, particularly in UC, while underscoring the critical importance of dose optimization and the need for validation in adequately powered clinical cohorts.

## Data Availability

The data supporting the findings of this study are available from the corresponding author upon reasonable request. The data are not publicly available due to privacy and ethical restrictions, as they were generated from human-derived biopsy samples and associated clinical information whose unrestricted sharing could compromise participant confidentiality.

## Abbreviations

The following abbreviations are used in this manuscript:

IBD	Inflammatory Bowel Disease
CD	Crohn’s Disease
UC	Ulcerative Colitis
SCFAs	Short-Chain Fatty Acids
CeMAD	Centro Malattie Apparato Digerente
IRCCS	Istituto di Ricovero e Cura a Carattere Scientifico
SES-CD	Simple Endoscopic Score for Crohn’s Disease
DMEM	Dulbecco’s Modified Eagle Medium
FBS	Fetal Bovine Serum
PBS	Phosphate-Buffered Saline
PET	Polyethylene Terephthalate
OCT	Optimal Cutting Temperature
BSA	Bovine Serum Albumin
5-ASA	5-Aminosalicylic Acid
H&E	Haematoxylin and Eosin
5-PL	Five-Parameter Logistic
ROI	Region of Interest
SD	Standard Deviation
ANOVA	Analysis of Variance
CTR	Control
TNF-α	Tumor Necrosis Factor alpha
IFN-γ	Interferon gamma
IL	Interleukin
IP-10 (CXCL10)	Interferon gamma-induced protein 10
MCP-1	Monocyte Chemoattractant Protein-1
MIP-1α	Macrophage Inflammatory Protein-1 alpha
MIP-1β	Macrophage Inflammatory Protein-1 beta
RANTES	Regulated upon Activation, Normal T cell Expressed and Secreted
G-CSF	Granulocyte Colony-Stimulating Factor
HDAC	Histone Deacetylase
